# Long noncoding RNA HCG18 up‐regulates the expression of WIPF1 and YAP/TAZ by inhibiting miR‐141‐3p in gastric cancer

**DOI:** 10.1002/cam4.3288

**Published:** 2020-07-29

**Authors:** Yan Liu, Wenji Lin, Yangyang Dong, Xinyu Li, Zhibin Lin, Jing Jia, Wenbing Zou, Yu Pan

**Affiliations:** ^1^ Department of Gastrointestinal Surgery (#2) Quanzhou First Hospital Affiliated to Fujian Medical University Quanzhou China; ^2^ Department of Radiology Quanzhou First Hospital Affiliated to Fujian Medical University Quanzhou China; ^3^ Department of General Surgery Fujian Medical University Union Hospital Fuzhou China

**Keywords:** HCG18, invasion, migration, miR‐141‐3p, viability

## Abstract

**Background:**

Accumulating works show that lncRNAs play critical roles in the development of gastric cancer (GC). LncRNA HLA complex group 18 (HCG18) was implicated in the progression of bladder cancer and glioma, but its role in GC is unknown.

**Methods:**

RT‐PCR was used to detect HCG18 and miR‐141‐3p expression in GC specimen. GC cell lines (AGS and MKN‐28) were exploited as cell model. The biological effect of HCG18 on cancer cells was probed by CCK‐8, colony formation, flow cytometry, Transwell and wound‐healing experiments in vitro, and subcutaneous xenotransplanted tumor model and tail vein injection model in vivo. Interaction between HCG18 and miR‐141‐3p was determined by bioinformatics analysis, RT‐PCR, and luciferase reporter experiments. Downstream gene expression of miR‐141‐3p, including Wiskott–Aldrich syndrome protein interacting protein family member 1 (WIPF1), Yes associated protein 1 (YAP), and tafazzin (TAZ) were detected using Western blot.

**Results:**

HCG18 was markedly up‐regulated in GC specimens, while miR‐141‐3p was markedly down‐regulated. Down‐regulation of HCG18 inhibited viability, migration, and invasion of GC cells, while miR‐141‐3p transfection led to opposite effect. HCG18 could down‐regulate miR‐141‐3p through adsorbing it, and a negative association between HCG18 and miR‐141‐3p was found in GC specimens. HCG18 promoted WIPF1, YAP and TAZ expression, nonetheless, such influence was reversed by co‐transfecting with miR‐141‐3p.

**Conclusion:**

HCG18 was aberrantly up‐regulated in GC tissues, and it indirectly regulated the activity of Hippo signaling through counteracting miR‐141‐3p expression.

## Graphical abstract

HCG18 was abnormally up‐regulated in GC tissues, and it could indirectly modulate the activity of Hippo signaling via reducing the expression level of miR‐141‐3p.

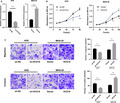

## 1 INTRODUCTION

One thing that is common to all is the fact that Gastric cancer (GC) is the prevailing gastrointestinal malignancy.^1^, ^2^ Although systemic combined therapies including surgical excision, radiotherapy, chemotherapy, and molecular targeted therapy have made great progresses, the therapeutic effect of GC patients is still less satisfying.^3^, ^4^, ^5^ Accumulating studies have investigated the molecular regulatory mechanism of GC, but the detailed molecular mechanisms in the occurrence and GC progression is still unknown.^6^

The studies in recent years have shown that abnormal expression of lncRNA links to the growth of multiple tumors.^7^, ^8^ Preceding researches have found that multiple lncRNAs are crucial regulators in GC. For instance, lncRNA HOTAIR facilitates GC cell proliferation (PLF), migration (MGT), and invasion (IVS) through targeting miR‐126, and in turn activating CXCR4 and RhoA Signaling Pathway.^8^ Identifying new carcinoma‐related lncRNAs has great implications for the diagnosis, therapy and prognosis prediction of GC.

MicroRNAs (miRNAs) are ncRNAs consisting of about 20 nucleotides, which can modulate multiple biological processes of cells.^9^ Recently, miR‐141‐3p has been shown to attach great importance to tumor development. For example, in GC, miR‐141‐3p can inhibit the expression of STAT4, to block the transformation from normal fibroblasts to carcinoma‐related fibroblasts.^10^ Notably, lncRNA can function as an endogenous competing RNA (ceRNA) to directly interact with miRNAs and modulate their expression and activity. For example, it has been discovered that lncRNA ATB targets miR‐200c to regulate the PLF and apoptosis (APS) of colorectal cancer cells.^11^ However, the ceRNA network in GC still needs to be explored.

The gene encoded by WASP and WIPF1 is implicated in the organization and aggregation of actin cytoskeleton, which is associated with cell PLF and IVS.^12^, ^13^ It has been experimentally demonstrated that WIPF1 is an oncogene in breast cancer, glioma, and colorectal cancer.^14^ Nonetheless, the function of WIPF1 in GC remains largely unknown. Furthermore, YAP and TAZ are key components of Hippo signaling pathway that consists of a cascade of kinases regulating cell PLF and differentiation.^15^ YAP/TAZ is highly expressed in some malignancies, such as GC, which is validated to modulate the organization of cytoskeleton and cell adhesion.^16^, ^17^ A recent study has found that WIP is able to promote tumor progression by regulating YAP/TAZ‐dependent autonomous cell development.^18^ Nevertheless, the roles of WIPF1 and YAP/TAZ in regulating GC and the specific mechanisms remain to be further studied.

The research was carried out to explore the expression, clinical implications, biological functions of HCG18 in GC and explore its downstream mechanisms. Herein, we demonstrated that HCG18 was up‐regulated in GC tissues, and linked to adverse prognosis of patients; additionally, it enhanced the malignant phenotypes of GC cells, and mechanistically, it functioned as a ceRNA to suppress miR‐141‐3p, and activating WIPF1/YAP/TAZ axis.

## 2 MATERIALS AND METHODS

### 2.1 Bioinformatics analysis

TCGA and GEPIA were exploited to detect HCG18 expression in various tumor tissues including GC and the correlation between HCG18 and WIPF1. Survival analysis was performed with K‐M plotter. LncBase Predicted v2 was used to predict the downstream target of HCG18. The binding sequence between miR‐141‐3p and WIPF1 was projected by TargetScan.

### 2.2 Sample collection

Our work was endorsed by the Ethics Committee. 79 GC patients undergoing surgery or biopsy from 2015 to 2019 were enrolled. Tumor tissues and normal paracancerous tissues were collected from them. The diagnosis of all the patients was validated by postoperative pathological examination. These patients were 26 to 70 years old, including 48 males and 31 females. None of them received chemotherapy or radiotherapy or took any targeted drugs before operation. Informed consent was signed by all the subjects. The tumor tissues and normal tissues collected were stored at −80℃.

### 2.3 Cell culture

Five GC cell lines (AGS, MKN‐28, NCI‐N87, MGC803 and SGC7901) and normal gastric mucosa epithelial cell GES1 were purchased from Aolu Biotechnology Co., Ltd. (Aolu, Shanghai, China). These cells were cultured in RPMI‐1640 medium or DMEM (Gibco, Carlsbad, CA, USA) supplemented with 10% FBS (Invitrogen, Carlsbad, CA, USA), 100 U/ml penicillin and 100 mg/ml streptomycin in an thermostatic incubator at 37℃/5% CO_2_ under saturated humidity.

### 2.4 Construction of cells

For the purpose of HCG18 overexpression, the DNA sequence encoding HCG18 was amplified with PrimeSTAR HS DNA polymerase (TaKaRa, Dalian, China) by PCR and cloned to pcDNA3.1 vector (Invitrogen, Carlsbad, CA, USA) to construct HCG18 overexpression plasmid. HCG18 shRNA (sh‐HCG18, 5'‐TTGGCTTCAGTCCTGTTCATCAG‐3') was synthesized by GeneChem (GeneChem, Shanghai, China). 5 × 10^4^ cells were inoculated to a 24‐well culture dish (1 ml/well). When the cells reached 70% confluence, Lipofectamine^®^ 3000 (Invitrogen, Carlsbad, CA, USA) was applied to transfect HCG18 overexpression plasmid or the negative control (vector) into MKN‐18 cells and sh‐HCG18 or the negative control (sh‐NC) into AGS cells under the guidance of the protocol. After 48 h of transfection, transfection efficiency was confirmed and cells were harvested for subsequent research. To establish AGS cells with stable HCG18 knockdown, lentivirus system carrying sh‐HCG18 (or sh‐NC) vectors was transfected into AGS cells, and puromycine (5 μg/ml) was used to screen the cells until the cell could stably proliferate. Then the cells were collected to detect HCG18 expression to validate the effect of knockdown.

### 2.5 qRT‐PCR

Total RNA was abstracted from GC cells with RNAiso Plus kit (Takara, Dalian, China). In order to detect miR‐142‐5p expression, total RNA was reversely transcribed with MMLV reverse transcriptase (Takara, Dalian, China). With the cDNA obtained as the template, RT‐PCR was executed with real‐time PCR kit (Qiagen, Shanghai, China). Then the miRNA expression was quantified by real‐time PCR and its relative level was normalized to U6. To detect HCG18 and WIPF1 mRNA expression, first‐strand cDNA was synthesized by using cDNA Synthesis Kit (Takara, Dalian, China) under the guidance of the protocol. PCR was executed by using SYBR greener qPCR SuperMix (Applied Biosystems, Shanghai, China). Relative expression was calculated using 2^‐△△Ct^ method. Table 1 shows the specific primer sequences.

**TABLE 1:** The primers used for qRT‐PCR

Name	Primer sequences
HCG18	Forward: 5’‐ATTCTCACTCTGGGGTTGGG‐3’
	Reverse: 5’‐TGATGTTGGCTGTGGGTTTG‐3’
WIPF1	Forward: 5’‐AATGGTGCCTTACTTTGTGATT‐3’
	Reverse: 5’‐TTTCTTCCTCTACGGTCCTTG‐3’
YAP	Forward: 5’‐CCACAGGCAATGCGGAATATC‐3’
	Reverse: 5’‐GGTGCCACTGTTAAGGAAAGG‐3’
TAZ	Forward: 5’‐GGCTGGGAGATGACCTTCAC‐3’
	Reverse: 5’‐AGGCACTGGTGTGGAACTGAC‐3’
GAPDH	Forward: 5’‐CATGAGAAGTATGACAACAGCCT‐3’
	Reverse: 5’‐AGTCCTTCCACGATACCAAAGT‐3’
miR‐141‐3p	Forward: 5’‐CAUCUUCCAGUACAGUGUUGGA‐3’
U6	Reverse: 5′‐CTCGCTTCGGCAGCACATATACT‐3′

### 2.6 Cell PLF assay

AGS or MKN‐28 cells were seeded to 96‐well plate (2 × 10^3^ cells/well). Each well was added with CCK‐8 kit (Dojindo, Kumamoto, Japan) (5 mg/ml) at 0 h, 24 h, 48 h and 72 h after transfection separately. Then the culture was continued in the incubator for 1 h. Then optical density (OD) value at 450nm was measured.

### 2.7 Transwell assay

For IVS experiment, before the experiments, the membranes of Transwell chambers (8 µm pore size, BD Biosciences, CA, USA) were paved with Matrigel^®^ (BD Biosciences, CA, USA) (80 μl/well) diluted with medium at a ratio of 1:8. For MGT experiment, Matrigel^®^ was not used. Medium containing 10% FBS was added into a 24‐well plate (700 μl/well), and then Transwell chamber was placed into each well of the 24‐well plate. Then successfully transfected cells suspended in serum‐free medium were inoculated into the upper compartment of Transwell chamber (2 × 10^5^ cells/compartment). After 24 h of culture, Transwell chambers were taken out, fixed in 4% paraformaldehyde solution for 10 min, and stained with 0.1% crystal violet solution for 30 min. Cells failing to pass through the chamber membrane were removed with cotton swab. Cells were counted under microscope to indicate their ability of MGT or IVS.

### 2.8 Luciferase reporter experiment

Luciferase reporter vector was established with DNA oligonucleotide and pMiR‐Reporter vector (pMiR‐HCG18‐WT/pMiR‐HCG18‐Mut) (Promega, Madison, WI, USA). pMiR‐HCG18‐WT or pMiR‐HCG18‐Mut together with miR‐141‐3p mimics or negative control (NC miR) were co‐transfected into AGS and MKN‐28 cells. Luciferase activity was measured by using dual‐luciferase reporter gene assay kit (Promega, Madison, WI, USA) 24h after transfection.

### 2.9 Western blot

RIPA lysis buffer containing protease inhibitor Leupeptin (Roche, Basel, Swizerland) was used to prepare CG cell lysis solution. The protein sample obtained was separated by SDS‐PAGE and transferred onto nitrocellulose (NC) membrane. After being blocked with 5% skim milk, the membrane was incubated at 4℃ together with primary antibodies, including anti‐WIPF1 (1:1000, Abcam, ab132512), anti‐YAP (1:1000, Abcam, ab52771), anti‐TAZ (1:1000, Abcam, ab84927), and anti‐GAPDH (1:1000, Abcam, ab141703). The membrane was washed with TBST and then incubated together with horse radish peroxidase (HRP) conjugated secondary antibody (1:2000, Santa Cruz Biotechnology) for 1 h. After the membrane was exposed with ECL chromogenic reagent (Millipore, Bedford, MA, USA), automatic imaging system (ChemiDocXRS imaging system) was used for image acquisition, and the gray level was calculated.

### 2.10 Tumorigenesis assay

All the animal assays were endorsed by the Institutional Animal Care and Use Committee. Female athymic BALB/c nude mice (4‐week old) were available from Shenzhen Topbiotech Co., Ltd. (Topbiotech, Shenzhen, China). These mice were randomly categorized into sh‐NC group (N = 10) and sh‐HCG18 group (N = 10). GC cells with HCG18 knockdown (sh‐HCG18) and negative control cells (sh‐NC) (1 × 10^7^ cells) were injected into the right abdomen of each mouse. Tumor size was measured every 7 days. All the mice were sacrificed on day 35 after injection, and xenotransplanted tumors were resected and weighted. Besides, WIPF1, YAP, and TAZ expression in tumor tissues were determined by qRT‐PCR and Western blot. In the lung metastasis (MTS) study, 1 × 10^7^ cells (sh‐NC group vs sh‐HCG18 group) were injected into caudal vein of 10 mice in each group. The mice were sacrificed and lung colonization was quantified through pathological examination after two weeks.

### 2.11 Statistical analysis

Statistical analysis was processed by GraphPad Prism 8.0 (GraphPad Prism Software, San Diego, CA, USA). Data were expressed as mean ± standard deviation (SD). Comparisons between different groups were executed by Student's t test or ANOVA. Numeration data were processed by chi‐square test. Pearson's correlation analysis was conducted to explore the relationships between different gene expression in GC specimens. *P* < .05 signified statistical significance.

## 3 RESULTS

### 3.1 The up‐modulation of HCG18 expression in CG tissues is linked to GC unfavorable prognosis

**FIGURE 1:**
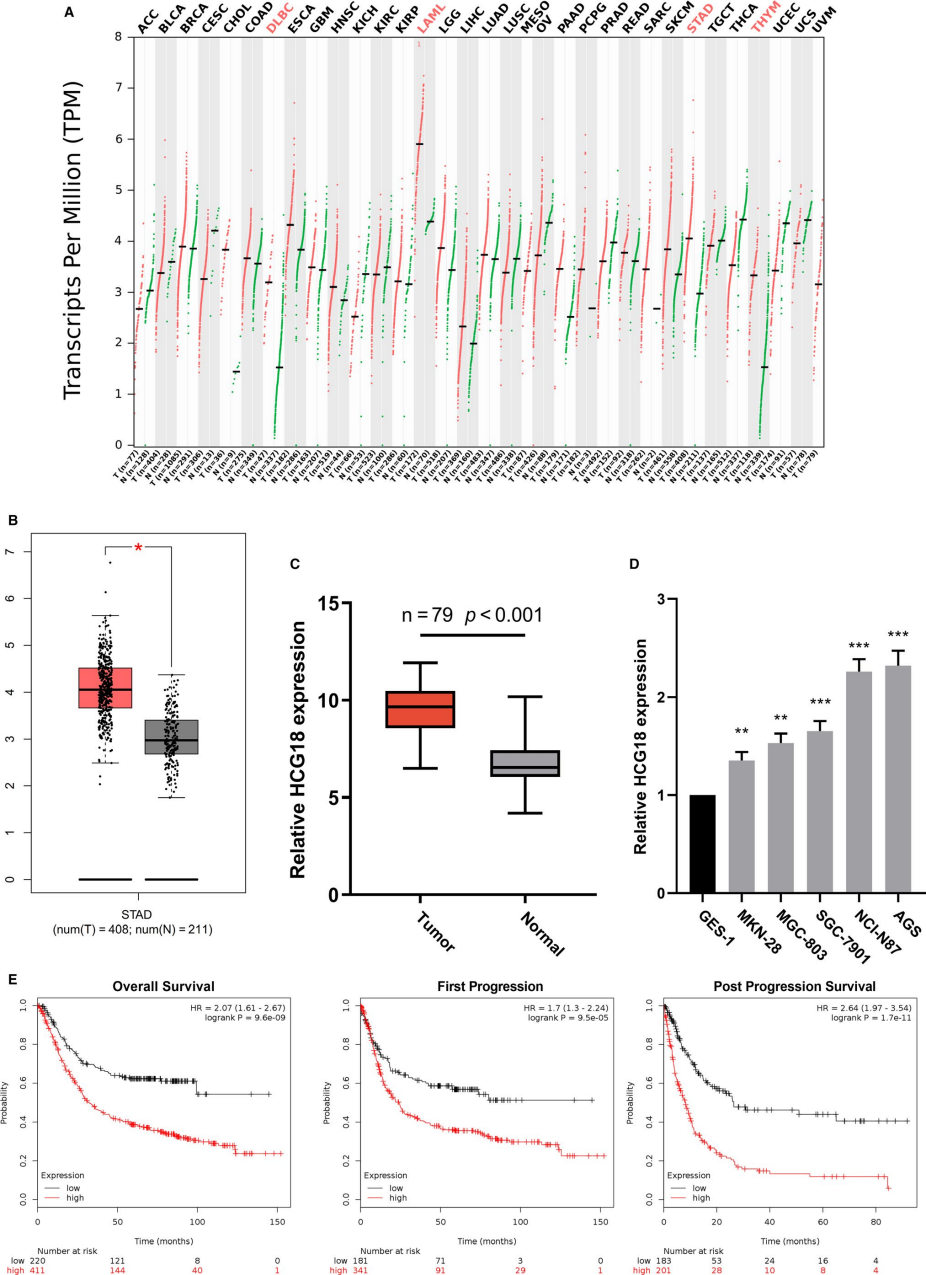
Increased HCG18 expression in GC tissues links to unfavorable prognosis. (A) HCG18 expressions in multiple cancers were obtained by searching GEPIA dataset (with TCGA data). (B) Different HCG18 expression in normal gastric tissues and GC tissues were obtained via searching GEPIA dataset (with TCGA data). (C) HCG18 expressions in 79 paired GC tissues and adjacent normal gastric tissues were detected using qRT‐PCR. (D) HCG18 expressions in normal gastric mucosa cell line and GC cell lines were detected using qRT‐PCR. (E) The connection for high HCG18 expression and the survival of GC patients was obtained via searching Kaplan Meier‐plotter database. ** *P* < .01, *** *P* < .001

According to TCGA, we searched HCG18 expression in multiple tissues and GC specimens and found that HCG18 was overexpressed in various cancer tissues in comparison with normal tissues especially in GC, which indicated tumor promoting role of HCG18 (Figure 1A and B). Besides, HCG18 expression was determined by qRT‐PCR in 79 GC specimens and adjacent tissue specimens. Similar to TGCA data, HCG18 expression in GC tissues was higher than adjacent normal tissue (Figure 1C). Furthermore, in comparison with normal gastric mucosa cell GES1, HCG18 expression in GC cell lines was higher (Figure 1D). To probe the correlation between HCG18 expression and patient's long‐term prognosis, the correlation between HCG18 expression and GC patient's prognosis was analyzed using K‐M plotter (http://kmplot.com/analysis/). The result indicated that high HCG18 expression was intimately linked to an unsatisfactory survival rate of GC patients (Figure 1E). The data implied that the up‐regulation of HCG18 expression attached great importance to GC development.

### 3.2 HCG18 expression linked to the clinicopathologic parameters of GC sufferer

To illustrate the function of HCG18 in the carcinogenesis and development of GC, we analyzed the association for HCG18 expression and diverse tumor pathological indicators based on the 79 GC specimens mentioned above (Table 2). The mean value of relative expressions of HCG18 in tumor tissues (qRT‐PCR data) was used as the threshold, and then the specimens were separated into high expression group or low expression group. Chi‐square test indicated that high HCG18 expression in tumor tissues was intimately associated with increased tumor size (*P = *.0165), local lymph node IVS (*P = *.0065), distant MTS (*P = *.0084), and increased of T stage (*P* = .0041), but had no obvious link with patient's age, gender, and degree of differentiation (*P* > .05), revealing that HCG18 may promote GC development.

**TABLE 2:** The relationship between HCG18 expression in 79 GC patients and clinicopathologic parameters

Clinicopathologic parameter	Total (n = 79)	HCG18 expression level		*P*‐value
		Higher (n = 41)	Lower (n = 38)	
Age (years)				
≤55	46	20	26	0.2164
>55	33	19	14	
Gender				
Male	48	25	23	0.5479
Female	31	14	17	
Tumor size				
<3 cm	43	13	30	0.0002
≥3 cm	36	26	10	
Histological grade				
High and middle	51	26	25	0.6987
Low	28	13	15	
Lymph node MTS				
No	49	16	33	0.0001
Yes	30	23	7	
T stage				
I/II	53	25	28	0.5770
III/IV	26	14	12	
Local IVS				
T1, T2	37	16	21	0.3069
T3, T4	42	23	19	

### 3.3 HCG18 regulated the PLF, MGT, and IVS of GC cells

**FIGURE 2:**
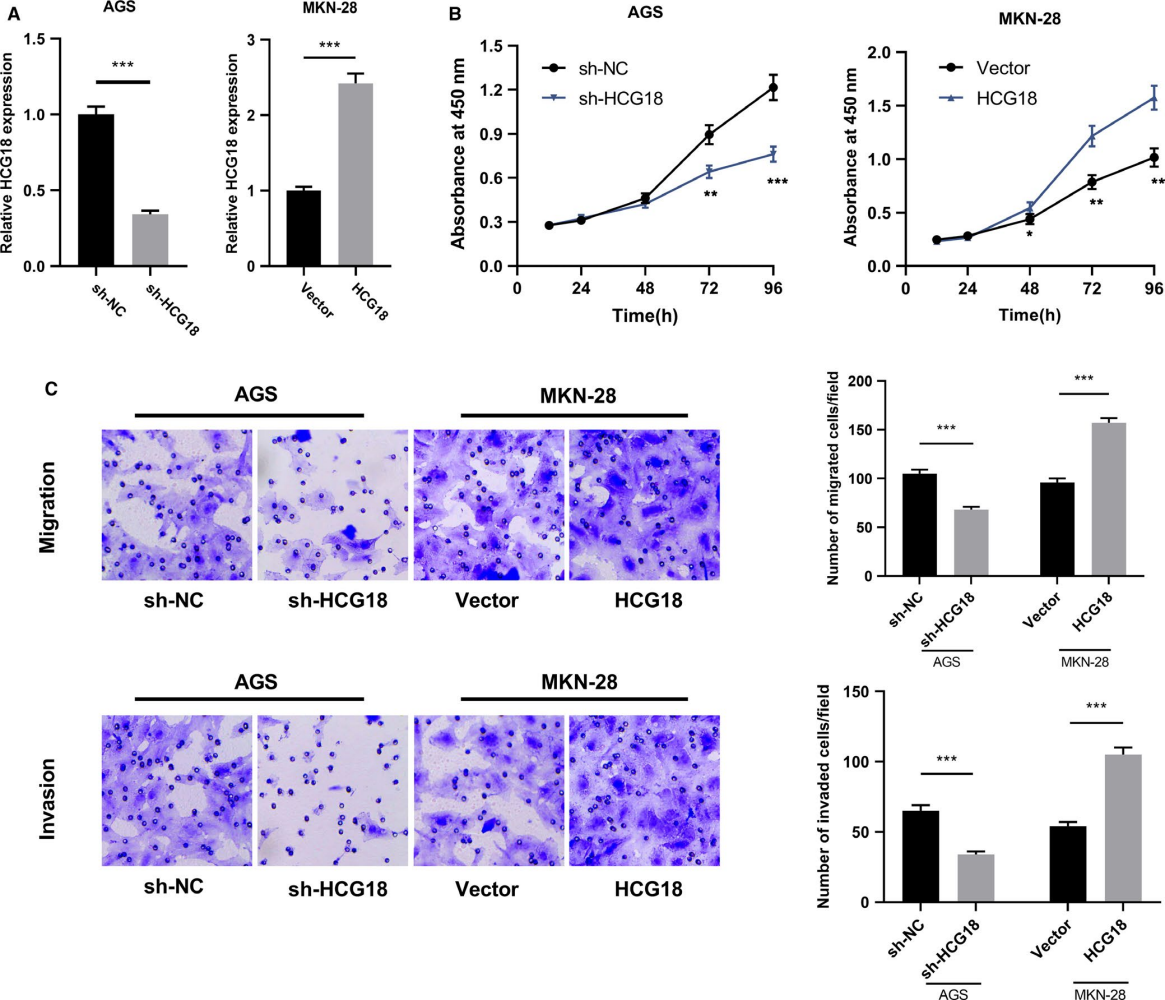
HCG18 can regulate the PLF, MGT and IVS of cells. (A) HCG18 expression in GC cells with HCG18 knockdown or high expression was detected using qRT‐PCR. (B) The PLF of GC cells with HCG18 knockdown or high expression was detected using CCK‐8 experiment. (C) The MGT and IVS of GC cells with HCG18 knockdown or high expression were detected using Transwell experiment. * *P* < .05, ** *P* < .01, *** *P* < .001

To better understand the biological functions of HCG18 in GC cells, AGS and MKN‐28 cells were used to construct a model of HCG18 knockdown and a model of HCG18 overexpression respectively (Figure 2A). Therefore, the PLF of these cells was detected using CCK‐8 experiment. The result revealed that in comparison with sh‐NC group, the PLF of AGS cells in HCG18 knockdown group was markedly suppressed, while the PLF of MKN‐28 cells in HCG18 overexpression group was remarkably enhanced (Figure 2B). Moreover, the influence of HCG18 on cell MGT and IVS were detected by Transwell experiment. The result showed that in comparison with sh‐NC group, the numbers of migrated cells and invasive cells in sh‐HCG18 group were remarkably reduced, while those in HCG18 overexpression group was evidently increased (Figure 2C). These findings suggest that HCG18 promotes the malignant phenotypes of GC cells.

### 3.4 The regulatory relationship between HCG18 and miR‐141‐3p

**FIGURE 3:**
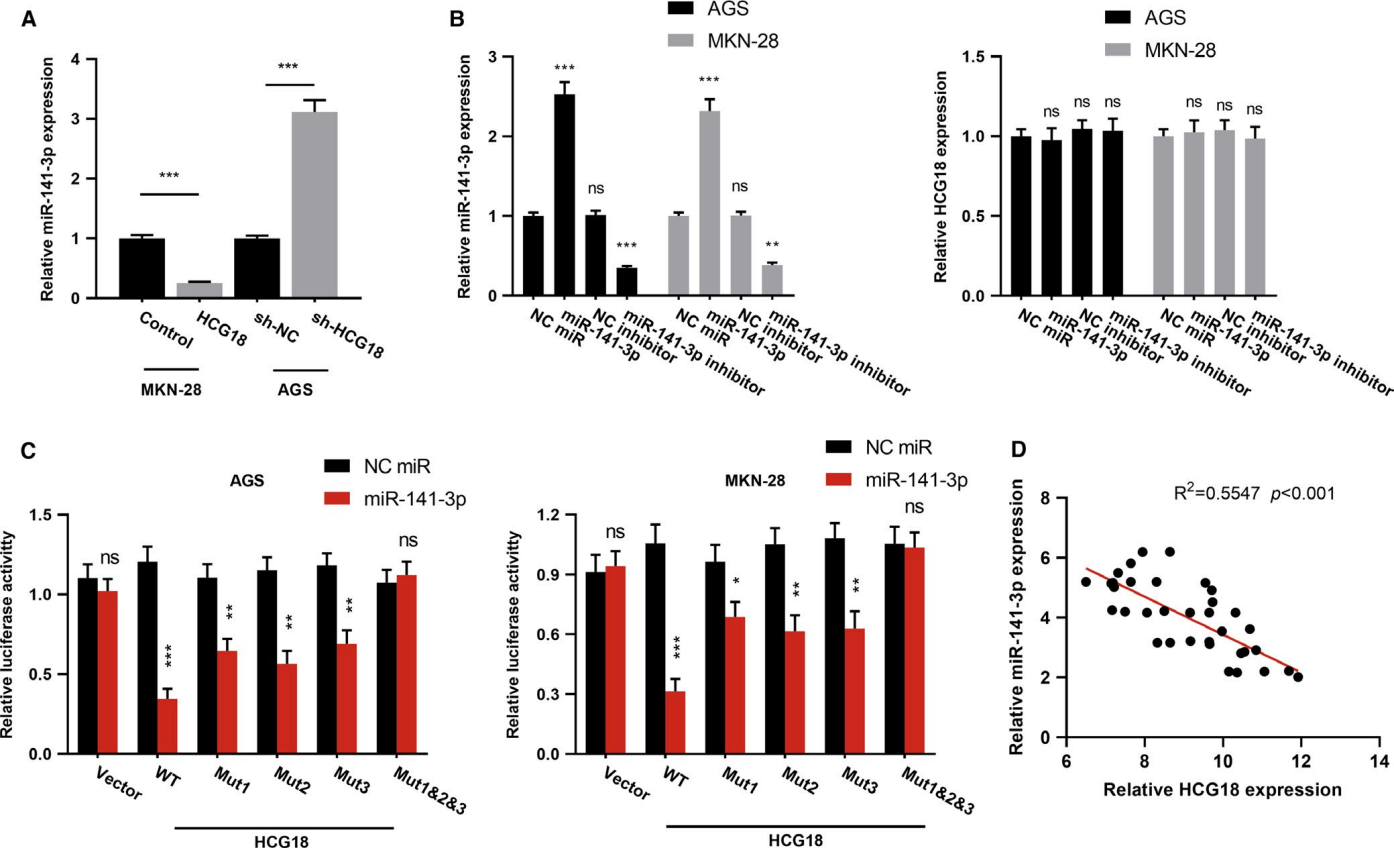
The regulation relationship for HCG18 and miR‐141‐3p. (A) miR‐141‐3p expression in GC cells with HCG18 knockdown or high expression was detected using qRT‐PCR. (B) miR‐141‐3p expression (left) and HCG18 expression (right) in GC cells after transfection of miR‐141‐3p mimics or inhibitors were detected using qRT‐PCR. (C) Targeting relationship between HCG18 and miR‐141‐3p in AGS (left) and MKN‐28 (right) cells were determined using dual luciferase activity experiment. (D) The correlation for HCG18 with miR‐141‐3p in clinical samples was validated using qRT‐PCR. * *P* < .05, ** *P* < .01, *** *P* < .001

To clarify the downstream mechanism of HCG18 in regulating the phenotypes of GC cells, we performed bioinformatics analysis with LncBase Predicted v2. Data show that HCG18 contains the binding sites for miR‐141‐3p (Figure S1). We further detected miR‐141‐3p expression in GC cell lines overexpressing and underexpressing HCG18 by qRT‐PCR, and found that miR‐141‐3p expression was decreased in cells overexpressing HCG18, but increased in cells with HCG18 knockdown (Figure 3A). We noticed that the transfection of miR‐141‐3p mimics or inhibitors did not change the expression level of HCG18 of GC cells (Figure 3B). Furthermore, miR‐141‐3p mimics weakened the luciferase activity of pMiR‐HCG18‐WT, but had no obvious influence on pMiR‐HCG18‐MUT (Figure 3C). The data inferred that miR‐141‐3p could directly combine with HCG18 at the recognition sites of microRNA. The relationship between HCG18 expression and miR‐141‐3p expression in GC tissues were further explored using qRT‐PCR, and the result denoted that HCG18 expression in GC specimens was negatively correlated with miR‐141‐3p expression (Figure 3D). The findings supported that miR‐141‐3p was a target of HCG18 in GC.

### 3.5 The effect of HCG18/miR‐141‐3p axis on cell PLF and MTS of GC cells

**FIGURE 4:**
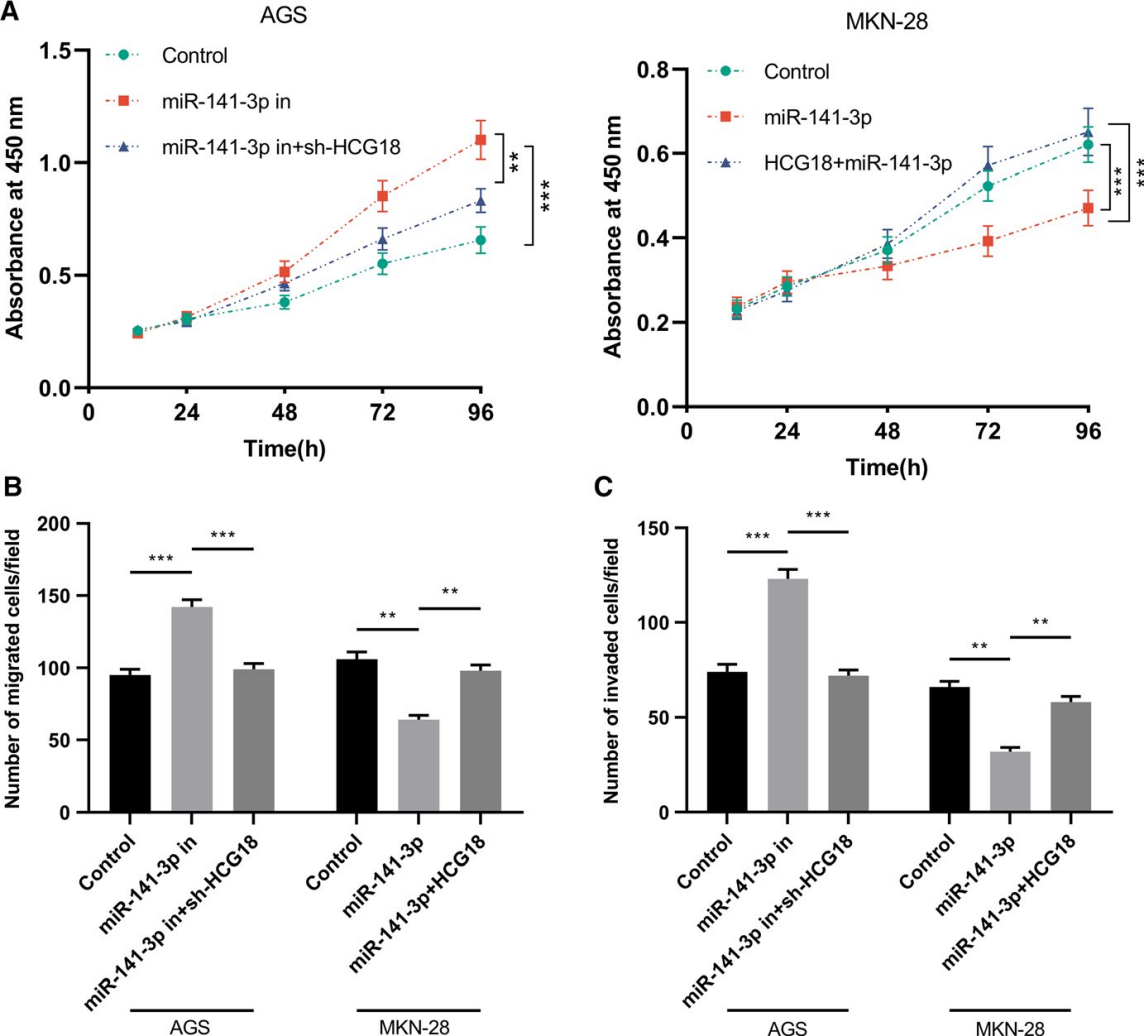
HCG18 knockdown offsets the enhancing effects of miR‐141‐3p inhibitor on cells. (A) The PLF of GC cells after co‐transfection of HCG18 shRNA/plasmids and miR‐141‐3p mimics/inhibitors were monitored using CCK‐8. (B and C) MGT (B) and IVS (C) of GC cells were detected using Transwell experiment after regulating miR‐141‐3p and HCG18 expression. ** *P* < .01, *** *P* < .001

To investigate the interaction between HCG18 and miR‐141‐3p, as well as their roles in regulating the progression of GC, miR‐141‐3p was highly expressed in GC cells overexpressing HCG18, and miR‐141‐3p was inhibited in GC cells with HCG18 knockdown. We found that miR‐141‐3p overexpression weakened the role of HCG18 overexpression in promoting malignant phenotypes, while miR‐141‐3p inhibition undermined the repression of the PLF, MGT, and IVS of GC cells by HCG18 knockdown (Figure 4A‐C). These results revealed that HCG18 promoted the PLF, MGT, and IVS of GC cells via modulating miR‐141‐3p.

### 3.6 HCG18 regulated WIPF1 and YAP/TAZ in GC cells by modulating miR‐141‐3p

**FIGURE 5:**
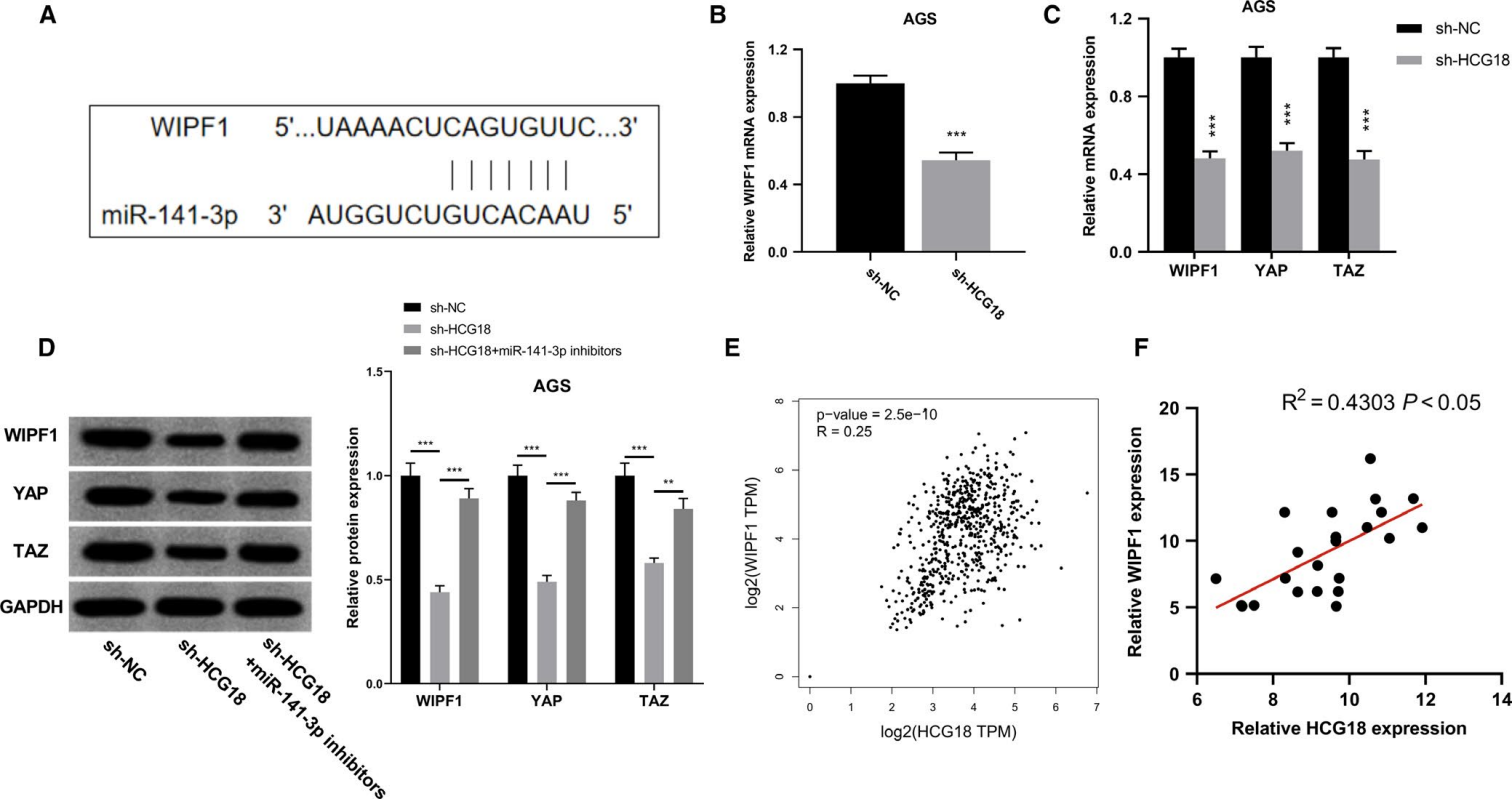
HCG18 regulates WIPF1 and YAP/TAZ in GC cells by modulating miR‐141‐3p. The bind sites for miR‐141‐3p and the 3’UTR of WIPF1 were predicted by TargetScan database. The regulatory effects of HCG18 on the mRNA expression of WIPF1 were detected by qRT‐PCR. (C) The regulatory effects of HCG18 on the mRNA expression levels of WIPF1, YAP/TAZ were detected using qRT‐PCR. (D) WIPF1, YAP/TAZ protein expressions were detected by Western blot after AGS cells were transfected with sh‐HCG18 and miR‐143‐3p inhibitors. (E) The correlation between HCG18 expression and WIPF1 expression was detected using TCGA data. (F) The link for HCG18 expression and WIPF1 expression in the GC samples collected was analyzed with qRT‐PCR data. ** *P* < .01, *** *P* < .001

After confirming that HCG18 modulated miR‐141‐3p expression, we tried to explore the downstream mechanism of miR‐141‐3p. Reportedly, WIPF1 is a target of miR‐141‐3p (Figure 5A),^19^ so we explored whether HCG18 was able to regulate WIPF1 expression. We found that HCG18 knockdown could significantly reduce the expression levels of WIPF1 and YAP/TAZ mRNA and protein (Figure 5B, C and D). Moreover, our results suggested that inhibiting HCG18 decreased WIPF1, YAP and TAZ expression and the phenomenon was reversed after transfection of miR‐141‐3p inhibitors (Figure 5D). According to the data of TCGA, we searched the connection for HCG18 and WIPF1 expression in GC tissues (Figure 5E). Consistent with the data of TCGA, the result of qRT‐PCR on the tissue samples suggested that HCG18 expression and WIPF1 expression were positively correlated in GC (Figure 5F). These results indicate that HCG18 can regulate the expressions of WIPF1, YAP, and TAZ in GC cells by modulating miR‐141‐3p.

### 3.7 WIPF1 regulated GC development

**FIGURE 6:**
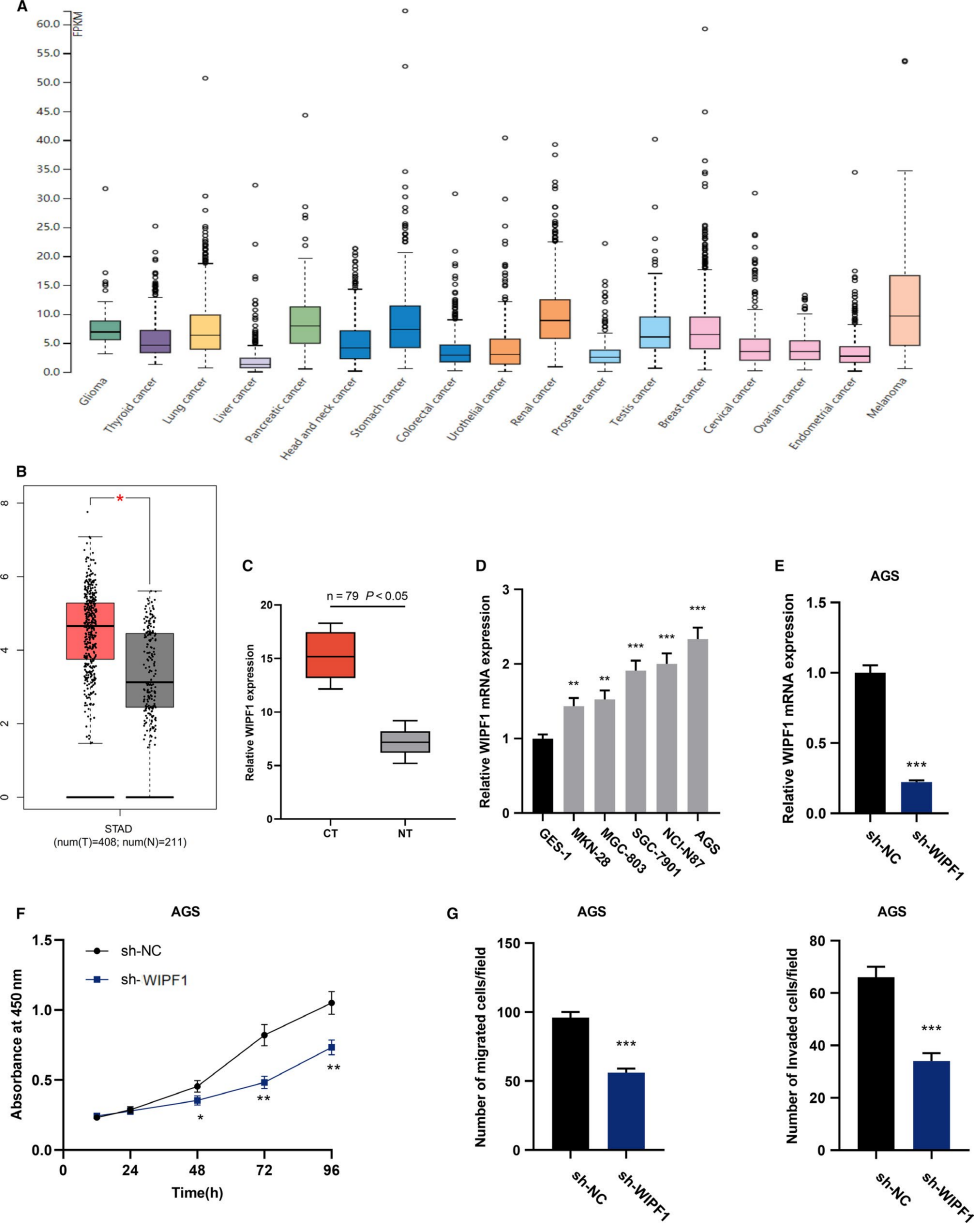
WIPF1 regulates the progression of GC. (A and B) WIPF1 expression in diverse cancers (A), such as GC (B), were obtained by searching GEPIA dataset (with TCGA data). (C) WIPF1 expression in the paired GC specimens and normal paracancerous tissues collected was detected using qRT‐PCR. (D) WIPF1 expression in GC cell lines were detected using qRT‐PCR. (E) WIPF1 expression was knocked down by shRNA in AGS cells, and WIPF1 mRNA expression was detected using qRT‐PCR. (F) PLF of AGS cells was monitored after WIPF1 was knocked down using CCK‐8 assay. (G) MGT (left) and IVS (right) of AGS cells were detected using Transwell experiment after WIPF1 knockdown. * *P* < .05, ** *P* < .01, *** *P* < .001

Due to the data of TCGA, WIPF1 expression in multiple tissues was searched, and demonstrated that WIPF1 was up‐modulated in GC tissues (Figure 6A and B). To validate whether HCG18 modulates the PLF and MTS of GC cells through miR‐141‐3p/WIPF1, different expression level of WIPF1 in GC cells was determined using qRT‐PCR. The result was similar to the data of TCGA: WIPF1 was overexpressed in GC (Figure 6C). In comparison with normal gastric mucosa cell GES‐1, WIPF1 mRNA expression was markedly elevated in GC cell lines (Figure 6D). Next, a cell model of WIPF1 knockdown was successfully established (Figure 6E). Then we detected the role of WIPF1 in GC. The PLF and MTS of GC cells were detected using CCK‐8 and Transwell experiments respectively. Our data indicated that WIPF1 knockdown could significantly impede the PLF and MTS of GC cells, suggesting that WIPF1 could enhance the biological functions of GC cells (Figure 6F and G).

### 3.8 Inhibiting HCG18 suppressed the growth and MTS of tumor in vivo

**FIGURE 7:**
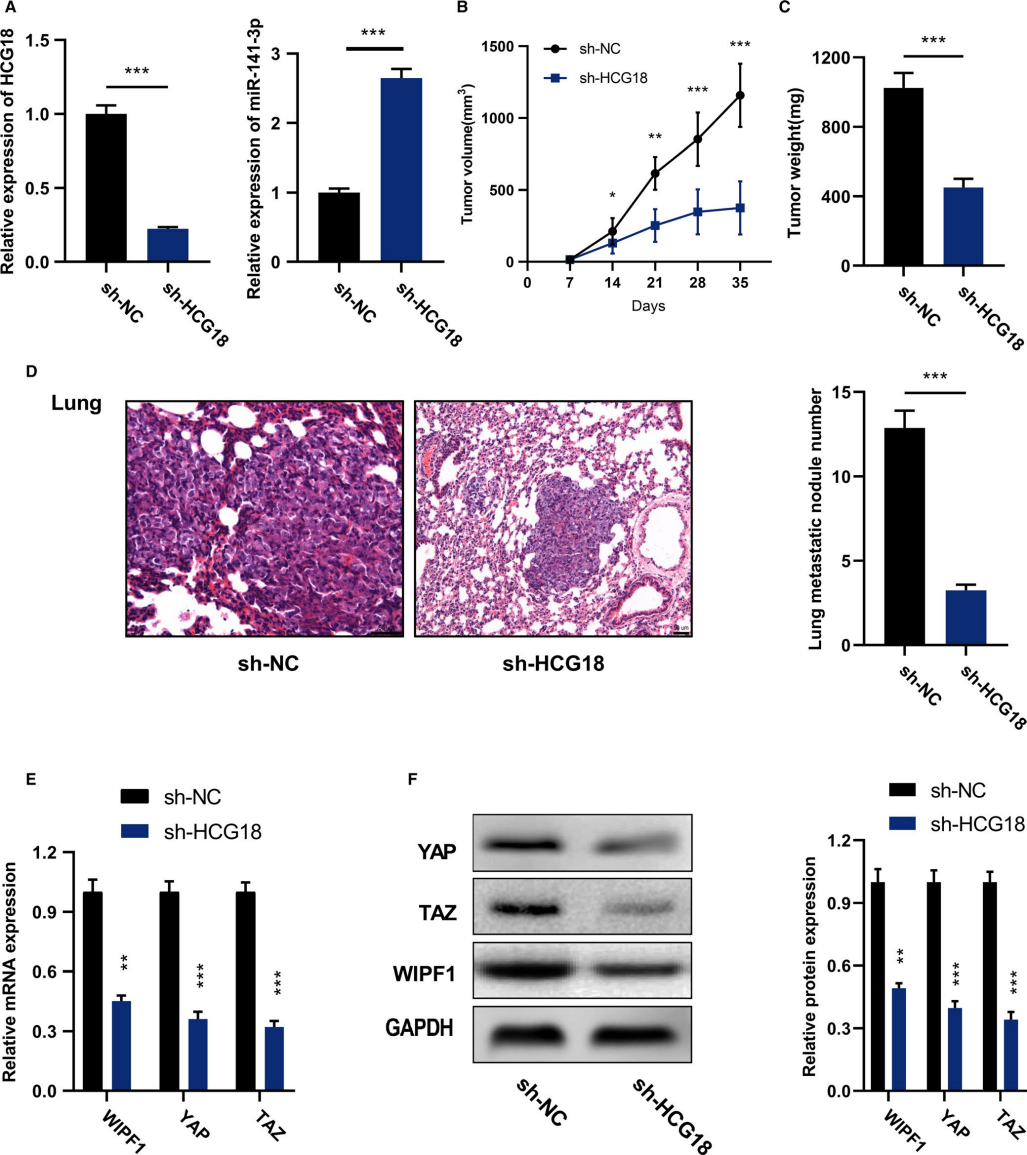
Inhibiting HCG18 suppresses the growth and MTS of tumor in vivo. (A) After the AGS cells with stable HCG18 knockdown were obtained, HCG18 expression (left) and miR‐141‐3p expression (right) were detected using qRT‐PCR. (B) The tumor size of the mice was detected every 7 days. (C) Weight of the tumors removed from the mice was measured. (D) The effect of HCG18 knockdown on tumor cells metastasizing to the lungs was evaluated. (E) The expressions of WIPF1, YAP, and TAZ in tumor tissues were detected using qRT‐PCR. (F) The protein expressions of WIPF1 and YAP/TAZ in tumor tissues were detected by Western blot. * *P* < .05, ** *P* < .01, *** *P* < .001

To probe whether HCG18 knockdown influences the growth and MTS of tumor in vivo, HCG18 was knocked down using lentiviral system in AGS cells, and two stable cell lines were established (known as AGS/sh‐NC cells and AGS/sh‐HCG18 respectively). HCG18 and miR‐141‐3p expression in the two cells were detected respectively (Figure 7 A). On this basis, subcutaneous tumorigenesis models were established. The result suggested that the tumor size of AGS/sh‐HCG18 cells was much smaller than that of AGS/sh‐NC cells (Figure 7B). When the tumors were weighted, it was found that the tumor weight of AGS/sh‐HCG18 cells was significantly lower than that of AGS/sh‐NC cells (Figure 7C). To understand the efficacy of HCG18 knockdown on tumor MTS, AGS/sh‐HCG18 and AGS/sh‐NC cells were transplanted to the caudal vein of mice. All the mice were slaughtered and pulmonary MTS was checked 14 d after the injection. In comparison with AGS/sh‐NC cells, the severity and number of pulmonary metastases in mice injected with AGS/sh‐HCG18 were significantly reduced (Figure 7D). Additionally, it was found that HCG18 knockdown significantly decreased WIPF1 and YAP/TAZ expression (Figure 7E and F). These results reveal that HCG18 knockdown can inhibit the development and MTS of GC through modulating miR‐141‐3p and WIPF1 in vivo.

## 4 DISCUSSION

LncRNAs exert important functions in tumorigenesis and tumor progression by acting as oncogenes or tumor‐inhibiting factors. For example, in head and neck squamous cell carcinoma, lncRNA MYOSLID facilitates cancer cell MGT via regulating the EMT process^20^; lncRNA SNHG10 promotes the MTS of liver cancer through positive feedback regulation of SCARNA13.^21^ Recent studies have discovered that HCG18 is highly expressed in intervertebral disc degeneration and promotes its development.^22^ Moreover, it is reported that HCG18 suppresses cell PLF and MGT in bladder carcinoma via modulating miR‐34c‐5p/NOTCH1 axis.^23^ A recent GC study demonstrates that HCG18 regulates the tumorigenesis via PI3K/Akt pathway.^24^ Thus, it can be seen that HCG18 exerts an important function in regulating the biological characteristics of tumors, but its function in human tumors is controversial. Our research is consistent with the previous report,^24^ which reveals that HCG18 expression in GC tissues was remarkably higher than normal paracancerous tissues, and highly expressed HCG18 was strongly linked to a larger tumor size, lymph node IVS, and distal MTS. Additionally, high HCG18 expression was linked to GC adverse prognosis and could be used as a prognosis predictive factor. Our study also found that HCG18 overexpression could significantly promote the PLF, MGT, and IVS of GC cells, while HCG18 knockdown could repress the PLF, MGT, and IVS in vitro. Our in vivo research validated that HCG18 knockdown suppressed tumor growth and distal MTS. The data further supported that HCG18 functioned as a cancer‐promoting factor in the progression of GC.

miRNAs are implicated in diverse cell processes, such as cell differentiation, PLF, and APS. For instance, miR‐181a targets IL‐2 to regulate the activation and differentiation of CD4 + T cells.^25^ miR‐21 enhances the PLF and MTS of breast cancer cells by targeting LZTFL1.^26^ In GC, miRNAs have been found to play various regulatory roles in cell PLF, MGT, multidrug resistance, etc For example, miR‐4268 represses the PLF of GC cells by targeting Rab6B^27^; miR‐107 modulates the growth and MGT of GC cells by down‐regulating FAT4 to activate PI3K‐AKT signaling pathway^28^; miR‐200c reverses the cisplatin resistance of GC by targeting NER‐ERCC3/4.^29^ Reportedly, miR‐141‐3p expression is down‐regulated in multiple tumors. For example, miR‐141‐3p expression is decreased in CRC, and miR‐141‐3p high expression can inhibit cell PLF and MGT.^30^ miR‐141‐3p suppresses cell PLF and enhances APS by targeting GLI2 in osteosarcoma.^31^ Importantly, miR‐141‐3p expression is down‐regulated during the progression of GC,^32^ expressing that miR‐141‐3p primarily exerts tumor‐suppressive effect in GC. Accumulating researches reveal that lncRNAs work on miRNAs via serving as ceRNA and competitively inhibit miRNAs as molecular sponges. For example, lncRNA GAS5 may play a role as a ceRNA of miR‐21^33^; lncRNA H19 enhances the IVS of glioma cells via decoying miR‐675.^34^ The function of HCG18 in bladder cancer as a ceRNA is proven recently.^23^ Here, we presumed that HCG18 might be a ceRNA in GC. In order to verify this hypothesis, bioinformatics analysis was made, and the data indicated that HCG18 contains latent binding sites for miR‐141‐3p. We proved that HCG18 negatively modulated miR‐141‐3p expression in GC cells. Additionally, HCG18 and miR‐141‐3p expression in GC tissues were negatively correlated. Moreover, our luciferase activity experiment confirmed the direct binding correlation between HCG18 and miR‐141‐3p. The data inferred HCG18 could sponge miR‐141‐3p in GC.

WIP family exerts important functions in the progression of human tumors.^35^ The overexpression of WIPF1, a key member of WIP family, can promote the IVS of papillary thyroid carcinoma cells.^36^ Lowly expressed WIPF1 in colorectal cancer patients indicates a better prognosis.^14^ These findings suggest that WIPF1 has the features of oncogene and attaches great importance to the development and MTS of tumors. The findings reveal that WIPF1 knockdown impedes the growth and MTS of GC cells, revealing that WIPF1 mainly works as an oncogene in GC. YAP and TAZ participate in the modulation of other essential signaling pathways such as Wnt/β‐catenin pathway, and may act as the common endpoint of multiple pathways resulting in cancer development.^18^ Similar to WIPF1, YAP/TAZ is highly expressed in multiple malignancies.^18^ According to previous studies, WIPF1 can enhance the stability of YAP/TAZ to stimulate the growth of tumor in pancreatic ductal adenocarcinoma.^18^ In the present work, WIPF1 was identified as a target gene in GC, which is consistent with the previous report.^19^ This inspired us to further explore whether HCG18 knockdown can inhibit tumorigenesis by regulating WIPF1 and YAP/TAZ expression. AS expected, our results suggested that HCG18 knockdown could reduce the expression levels of WIPF1 and YAP/TAZ, and this effect could be reversed by miR‐141‐3p inhibition. These data suggested that at least partially, HCG18 exerted its biological effects by modulating miR‐141‐3p/WIPF1/YAP/TAZ pathway.

In conclusion, our study has found that HCG18 may promote the expression of WIPF1 and YAP/TAZ via repressing miR‐141‐3p and thus facilitate the PLF and MGT of GC cells. With the deepening of relevant studies in the future, HCG18 may become a prognostic biomarker and a therapeutic target for GC patients.

## Supporting information


**FIGURE S1:**
