# Supplementary figures and images for: Long noncoding RNA HCG18 up‐regulates the expression of WIPF1 and YAP/TAZ by inhibiting miR‐141‐3p in gastric cancer
Source: Cancer Med. 2020 Jul 29;9(18):6752–65. doi: 10.1002/cam4.3288 (PMC7520348; doi:10.1002/cam4.3288)

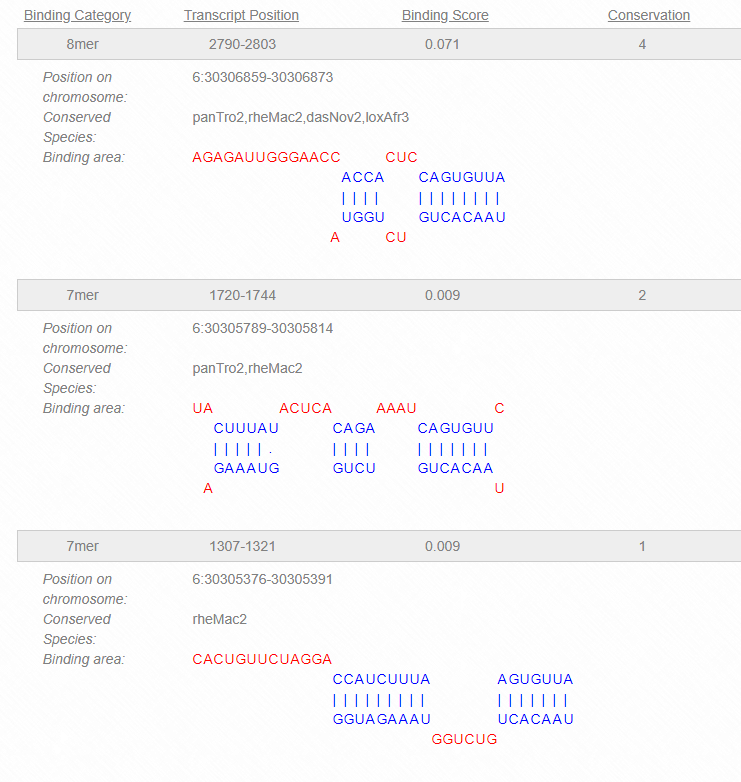

Supplement: Supplementary file 1 — FIGURE S1: [file CAM4-9-6752-s001.tif]
